# Malaria situation in a clear area of Iran: an approach for the better understanding of the health service providers’ readiness and challenges for malaria elimination in clear areas

**DOI:** 10.1186/s12936-020-03188-7

**Published:** 2020-03-18

**Authors:** Hosein Azizi, Elham Davtalab-Esmaeili, Mostafa Farahbakhsh, Maryam Zeinolabedini, Yagoub Mirzaei, Mohammad Mirzapour

**Affiliations:** 1 Department of Epidemiology and Biostatistics, School of Health, Tehran University of Medical Sciences, Tehran, Iran; 2 Research Center of Psychiatry and Behavioral Sciences, Tabriz University of Medical Sciences, Tabriz, Iran; 3 Basic Sciences Department, School of Allied Medical Sciences, Tabriz University of Medical Sciences, Tabriz, Iran; 4 Province Heath Center for Disease Control, Department of Communicable Diseases, Tabriz University of Medical Sciences, Tabriz, Iran; 5 Health Network Manager, Tabriz University of Medical Sciences, Tabriz, Iran

**Keywords:** Challenges, Elimination, Iran, Malaria, Readiness, Health service provider

## Abstract

**Background:**

Malaria mortality and morbidity have decreased in recent years. Malaria elimination (ME) and effective efforts to achieve ME is one of the most important priorities for health systems in countries in the elimination phase. In very low transmission areas, the ME programme is faced with serious challenges. This study aimed to assess the trend while getting a better understanding of Health Service Providers’ (HSPs) readiness and challenges for ME in a clear area of Iran.

**Methods:**

This study was performed in two phases. At first, the malaria trend in East Azerbaijan Province, was surveyed from 2001 to 2018; afterward, it was compared with the national situation for a better understanding of the second phase of the study. Data were collected from the Ministry of Health’s protocol and the health centre of the province. In the second phase, malaria control programme experts, health system researchers, and health managers’ opinions were collected via in-depth interviews. They were asked regarding HSPs readiness and appropriate Malaria Case Management (MCM) in a clear area and possible challenges.

**Results:**

A total of 135 and 154,560 cases were reported in the last 18 years in East Azerbaijan Province and Iran, respectively. The incidence rate decreased in East Azerbaijan Province from 0.4/10,000 in 2001 to zero in 2018. Furthermore, no indigenous transmission was reported for 14 years. Also, for the first time, there was no indigenous transmission in Iran in 2018. The main elicited themes of HSPs readiness through in-depth interviews were: appropriate MCM, holistic and role-playing studies for assessment of HSPs performance, system mobilization, improving identification and diagnosis of suspected cases in the first line. Similarly, the main possible challenges were found to be decreasing health system sensitivity, malaria re-introduction, and withdrawing febrile suspected cases from the surveillance chain.

**Conclusion:**

Health systems in eliminating phase should be aware that the absence of malaria cases reported does not necessarily mean that malaria is eliminated; in order to obtain valid data and to determine whether it is eliminated, holistic and role-playing studies are required. Increasing system sensitivity and mobilization are deemed important to achieve ME.

## Background

According to the World Health Organization (WHO) report in 2018, malaria mortality and morbidity have decreased in recent years [1]. In 2017, the number of malaria deaths was reduced from 607,000 to 435,000 compared to 2010, also, during these years the global incidence rate of malaria reduced from 72 to 59 cases per 1000 population at risk [2]. Many countries in the world made substantial progress in malaria elimination (ME). The WHO has defined that ME is the interruption of indigenous transmission of a specified malaria parasite species in a defined geographic area; continued measures are required to prevent re-establishment of transmission [3, 4].

In Iran, during the last decades, malaria has been the most important infectious disease with about 1500 cases per year, especially, in southern and southeastern areas [5, 6]. ME was started with political commitment, intersectional collaboration, and evidence-based research in 2009 and Iranian health system is planning for ME certification in 2025 [7]. During the last decades, vector control interventions as well as many effective efforts have been done for malaria control including enlarged coverage of integrated vector management (IVM), using rapid diagnostic tests (RDTs), appropriate case management, training of microscopists and Health Service Providers (HSPs), and developing a malaria early warning system [7–9]. For the first time, zero indigenous case reported in Iran in 2018. A large amount of effective efforts, environmental measures, and proper case management were conducted for malaria control in Iran [10].

In clear areas and countries in elimination phase, system readiness, an appropriate malaria case management (MCM), such as identification, diagnosis, appropriate treatment and urgent report of malaria cases to the public sector, and system vigilance were decreased [11]. In these areas, the management of suspected malaria or febrile cases is defective due to the very low or absence of malaria transmission [12, 13]. Therefore, the sustainability of elimination status and appropriate MCM are necessary for pre-elimination. Based on the province malaria department data in northwest of Iran, East Azerbaijan Province, the indigenous transmission has been absent for 14 years and imported cases were very few. However, it seems that in clear areas, malaria surveillance and HSPs readiness statuses are poorly understood. Thus, advances in understanding local and up-to-date information about malaria trends and situations in clear areas are essential for the National Malaria Control Programme and health policymakers in countries in the elimination phase. Another aim of this study is to assist policymakers in better understanding of system readiness and challenges for ME as well as adopting an appropriate strategy to adjust barriers and challenges in clear areas [14]. Hence, the present study aimed to investigate HSPs readiness and challenges for ME and malaria trends in East Azerbaijan Province from 2001 to 2018.

## Methods

### Study design

This study was performed in East Azerbaijan Province, northwest of Iran in which no Indigenous malaria case has been reported for 15 years (from 2005). This study was composed of two sections. In the first section, malaria trend was investigated from 2001 to 2018 in East Azerbaijan Province, an area of Iran considered cleared of malaria.

In section two, 6 in-depth interviews were conducted with two malaria field experts (one with master’s degree and the other a medical doctor), who monitored and supervised malaria control programme, two were vice managers and the other two were researchers with PhD in health system from Tabriz University of Medical Sciences. In Iran, medical universities are responsible authorities for the health system in each province. The Iranian health system is composed of three levels based on referral system; the first level is primary level of health services to access all urban and rural populations by family physicians and various types of HSPs. When patients need special health services, they are referred to the second level (county hospitals) by family physicians, and when high special services are required, patients are referred to province referral hospitals with high special services [15, 16]. It is worthwhile to mention that in Iran all malaria control services in the public sector such as diagnosis and treatment of malaria cases are free and anti-malarial drugs are available only in the public sector in the first referral level. The public sector is responsible for malaria control in Iran. Not to mention that private sector has to report any identification of a suspected malaria case to public sector.

### Definitions

*Health service providers (HSPs)* are primary health care practitioners who visit people with common medical problems. HSPs are the first line providers whom suspected malaria cases referred in the first step. These providers comprised of emergency physicians, family physicians, general practitioners, public or private sector specialists (in health centres, hospitals, and clinics), pharmacists, health workers, and health care providers in cities and villages.

*HSPs readiness* in this study, readiness is relevant to HSPs practice and performance in the management of patients with a history compatible with suspected malaria. HSPs readiness is a part of health system readiness, and the aim of this study was an appropriate MCM by HSPs including identification of suspected malaria cases, correct diagnosis, prompt and appropriate treatment according to the national guideline and urgent report to the public sector.

*Role-playing method* in the elimination phase, the malaria cases are rare or absent. Therefore, HSPs cannot demonstrate their actual performance for real malaria cases. Adjusting this barrier could be obtained through using artificial patients or standard cases in a role-playing design as a fake suspected malaria case. Accordingly, role-playing is the changing of one’s behaviour to assume a suspected malaria case role to assess HSPs performance in a realistic situation.

*HSPs mobilization* is a capacity-building process, encouragement and pro-activating HSPs in the case management of suspected malaria cases to improve the identification, early diagnosis, prompt and appropriate treatment, and control of malaria cases to quickly achieve the goal of elimination.

*Imported case* malaria case or infection in which the infection was acquired outside the area in which it is diagnosed.

*Indigenous case* A case contracted locally with no evidence of importation and no direct link to transmission from an imported case.

### Data collection

#### Malaria situation in East Azerbaijan, a clear area

The trend of malaria cases was obtained from the Ministry of Health’s protocol and provincial malaria control department and center for disease control. Provincial Health Center is a deputy of health in Tabriz University of Medical Sciences in East Azerbaijan and is responsible for the malaria control programme among all urban and rural areas by many types of HSPs, laboratories and environmental efforts. Malaria trend and malaria situation in East Azerbaijan Province were compared with the national situation for a better understanding of the Iranian malaria situation and malaria elimination programme goal with planning for elimination in 2025 and receiving ME certification which is awarded for national geographical level. Fortunately, in most Iranian provinces, there is no indigenous malaria transmission. Hence, the main objective of this study is to assess the HSPs readiness for ME in specific regions (East Azerbaijan Province) of Iran and to understand the challenges associated with ME in general.

#### In-depth interviews

The face to face in-depth interviews were conducted with 6 participants. The interviews, lasting at least 1 h, were audio-recorded and transcribed by two authors of the present study; subsequently, the results were compared and scrutinized separately. Prior to using questions for in-depth interviews, critical comments from three high experts were obtained (a health worker, a researcher, and an epidemiologist). Some questions were revised. The interviews comprised two main parts including many more in-depth questions. The first part was related to participants’ opinions regarding HSPs readiness for ME, HSPs performance in the case management of suspected malaria, and the participants’ prediction and inference about a real MCM status by HSPs in clear areas. The second part focused on possible challenges encountered by provincial health services in the elimination phase.

Furthermore, the following detailed, as well as ad hoc questions according to the interviewee’s answers and their level of expertise were asked under the first part framework. (1) How much do you think that the province’s health system is ready for ME? (2) How much HSPs are ready? (3) Do you think if a real febrile malaria patient were referred to providers, MCM would be done correctly? (4) What are the main weaknesses in the appropriate MCM? (5) Does the absence of local malaria cases provide evidence for local elimination? (6) What would be your recommendations to enhance system vigilance and HSPs Readiness?

Under the framework of second part, following questions were asked: (1) what do you think about ME challenges in the province? (2) Which major challenge do you think is the most critical? (3) What efforts should be performed for the stability of elimination status? (4) Do you think the health system is adequately alert and there is no concern for the re-introduction of malaria? (5) Is the health system and HSPs mobilized for ME? (6) Are there enough literature and studies about ME available?

#### Analysis and interpretation of interviews

After audio-recording and transcribing the interviews, the audio-recorded files were listened carefully and transcripts were written in Persian by two authors. Afterwards, the main themes, topics, and terms were extracted in each question and summarized in a brief explanation and interpretation by two experts. Interviews were read and compared line by line and then divided into categories. The core variable and main themes were extracted by the comparison between categories. In cases of misunderstanding, a member check was used. The transcripts were then translated into English by two experts while the names of interviewees were not indicated.

## Results

Table 1 illustrates the malaria incidence between national status and East Azerbaijan Province, a clear area of Iran, during 18 years from 2001 to 2018. The number of malaria cases decreased over the study period both in-country and province, but the incidence rate in East Azerbaijan Province is lower than that in the rest of the country.

**Table 1:** Trend of total malaria and Indigenous cases in Iran and East Azerbaijan province, 2001–2018

Years	East Azerbaijan			Iran		
	Incidence rate^b^	Total malaria cases	Indigenous cases	Incidence rate^b^	Total malaria cases	Indigenous cases
2001	0.4	40	19	3.1	19,129	^a^
2002	0.23	25	12	2.4	15,378	^a^
2003	0.3	35	29	3.8	25,027	^a^
2004	0.12	16	12	1.8	13,166	^a^
2005	0.04	6	0	2.8	19,285	14,634
2006	0.019	3	0	2.3	15,869	12,792
2007	0.018	2	0	2.3	16,489	12,538
2008	0.0063	1	0	1.6	11,333	6906
2009	0.02	4	0	0.8	5900	3606
2010	0	0	0	0.4	2965	1362
2011	0.0054	2	0	0.4	3108	1240
2012	0	0	0	0.2	1348	540
2013	0	0	0	0.2	1390	399
2014	0	0	0	0.16	1230	246
2015	0	0	0	0.1	777	156
2016	0	0	0	0.1	704	82
2017	0.0025	1	0	0.12	890	63
2018	0	0	0	0.07	572	0
Total		135	72		154,560	54,564

^a^Data not available

^b^Per 10,000 persons at risk

A total of 135 and 154,560 cases were reported during these years in East Azerbaijan Province and Iran, respectively. The indigenous transmission was zero from 2005 to 2018 in the province while in the whole country, it was zero for the first time in 2018. Most malaria cases occurred in province and whole country were reported in 2001 and 2003, respectively.

Among East Azerbaijan Province counties, Kaleybar had the highest infection rate with 72 cases during the years of study (Table 2). Out of 135 malaria cases in the province, 86.67% and 51.11% were men and aged 31–40 years, respectively. Almost, 86% of malaria patients in province lived in rural areas and 63% of them were laborer. Furthermore, *Plasmodium vivax* was the dominant parasite species with 83.7% (Table 3).

**Table 2:** Distribution of malaria cases in counties of East Azerbaijan province, Iran, 2001–2018

County	Years																	
	2001	2002	2003	2004	2005	2006	2007	2008	2009	2010	2011	2012	2013	2014	2015	2016	2017	2018
Azarshahr	0	0	0	0	0	0	0	0	0	0	0	0	0	0	0	0	0	0
Osku	0	0	0	0	0	0	0	0	0	0	0	0	0	0	0	0	0	0
Ahar	2	0	0	0	1	0	0	0	0	0	0	0	0	0	0	0	0	0
Ajabshir	0	0	0	0	0	0	0	0	0	0	0	0	0	0	0	0	0	0
Bostanabad	0	0	0	1	0	0	0	0	0	0	0	0	0	0	0	0	0	0
Bonab	0	0	0	0	1	0	0	0	0	0	0	0	0	0	0	0	0	0
Charoymaq	0	0	0	0	0	0	0	0	0	0	0	0	0	0	0	0	0	0
Jolfa	0	0	0	0	0	0	0	0	0	0	0	0	0	0	0	0	0	0
Sarab	0	0	0	0	0	0	0	0	0	0	0	0	0	0	0	0	0	0
Shabestar	0	0	0	0	0	0	0	0	0	0	0	0	0	0	0	0	0	0
Tabriz	0	0	0	0	0	0	1	1	0	0	0	0	0	0	0	0	0	0
Kaleybar	19	12	29	12	0	0	0	0	0	0	0	0	0	0	0	0	0	0
Maragheh	0	0	0	0	0	1	0	0	0	0	0	0	0	0	0	0	0	0
Marand	2	1	0	0	1	0	0	0	0	0	0	0	0	0	0	0	0	0
Malekan	1	2	1	2	0	1	0	0	0	0	1	0	0	0	0	0	0	0
Miyaneh	15	9	0	1	0	0	0	0	2	0	0	0	0	0	0	0	0	0
Hashtrud	1	0	0	0	0	0	1	0	0	0	0	0	0	0	0	0	0	0
Heris	0	0	0	0	0	0	0	0	0	0	0	0	0	0	0	0	1	0
Varzeqan	0	0	0	0	0	0	0	0	0	0	0	0	0	0	0	0	0	0
Total	40	25	35	16	6	3	2	1	4	0	2	0	0	0	0	0	1	0

**Table 3:** Demographic characters of malaria cases in East Azerbaijan, Iran, 2001–2018

Variables	N = 135	%
Gender		
Female	18	13.33
Male	117	86.67
Age groups		
≤ 20	8	5.92
21–30	46	34.07
31–40	69	51.11
> 40	12	8.88
Residence		
Urban	19	14.07
Rural	114	85.93
Occupation		
Laborer	85	62.96
Housewife	18	13.33
Farming or farming related	9	6.66
Others	23	17.04
Parasite species		
*P. vivax*	113	83.7
*P. falciparum*	20	14.81
*P. malariae*	0	0
*P. vivax *+* P. falciparum*	2	1.48

Table 4 demonstrates malaria screening (case finding) trend by primary health care (PHC) in East Azerbaijan Province during 2004–2018. A total of 46,304 microscopic slides were taken and tested during the years of the study. The findings indicated that the numbers of suspected screened cases decreased whereas slide examination (reading) in less than 48 h (the time interval between preparation and microscopic examination increased from 2004 to 2018.

**Table 4:** Malaria screening (case finding) trend in East Azerbaijan province by primary health care, 2004–2018

Years	Numbers of slides^a^	screening rate/1000	Slide examination less than 48 h (%)^b^
2004	7412	5.5	33
2005	5638	3.88	34.5
2006	4778	3.02	35.8
2007	3415	1.75	39.7
2008	2460	1.1	41.5
2009	2339	0.78	43.8
2010	1739	0.49	56.9
2011	1631	0.44	57.4
2012	2013	0.53	73.8
2013	2366	0.71	84
2014	2604	0.68	87
2015	2427	0.63	90
2016	3007	0.77	87
2017	2623	0.67	90.2
2018	1852	0.46	89
Total	46,304	1.427 (mean)	62.90 (mean)

^a^Slides were taken from febrile cases or subjects how had travel history to the endemic areas

^b^Slide examination is a period between slide preparation and lab investigation

Table 5 summarizes the main topic and themes of two interview parts respecting HSPs readiness and MCM in a clear area as well as possible challenges in the elimination phase. According to the recorded interviews and extracted results and interpretation, the following themes and categories were elicited.

**Table 5:** Extracted main topic and themes from in-depth interviews in relation to HSPs readiness and challenges for malaria elimination in a clear area of Iran

Topics of in-depth interviews	The main themes
Part 1) Health service providers readiness	Inappropriate malaria case management
	Low holistic and role-playing studies about system readiness and health service providers performance in clear areas
	Quality of malaria identification and diagnosis
	Need to health worker re-training, system mobilization and support
Part 2) Challenges of clear areas in elimination phase	Decreasing of health system sensitivity due to absent of malaria cases
	Possibility of malaria re-introduction and neighbor countries with endemic situation
	Exiting febrile suspected cases from system surveillance chain

### HSPs readiness and malaria case management

#### Appropriate malaria case management

According to the participants’ views, if a real case of malaria refers to HSPs, it may not be managed correctly. Participants pointed out that there were many criteria for correct MCM including eliciting travel history from febrile cases, requesting appropriate diagnosis tests, providing appropriate treatment in less than 12 h and preparing urgent report to public health sector. Also, participants expressed their concerns regarding febrile suspected MCM based on assessed knowledge and practice of HSPs in the field. Besides, the interviewees asserted that when febrile suspected malaria referred to HSPs, most of the time, they were not assessed for malaria and there existed several shortcomings in the process of identifying patients with suspected malaria. As a result, sometimes, febrile cases were treated for endemic febrile diseases without being assessed for malaria morbidity in the first place.

#### Holistic and role-playing studies

Interviewees believed that holistic, role playing, and KAP studies were required to evaluate HSPs performance, practice, attitude, and knowledge in the management of febrile suspected malaria cases in clear areas. Due to the absence of a real malaria patient in the elimination phase, performing a study through using a role-playing technique could provide better understanding of the disease control situation according to participants.

#### Malaria identification and diagnosis

In addition, interviewees commented on the quality of blood smears has been deteriorated over the last years. Sometimes, a thin and a thick spread of smears by first line HSPs were not acceptable. Based on patient flow, RDTs and anti-malarial drug availability should be emphasized and considered important in sections with higher possibility of suspected malaria cases such as emergency departments, clinics, and public sectors.

#### System mobilization

Most of the participating experts highlighted the need to a capacity-building process for system and HSPs mobilization in clear areas. In order to access ME criteria, the following procedures should be put into schedule: encouragement and mobilization of the health system and HSPs, training and re-training programmes, strengthening system support, and intersectional collaborations.

### Malaria elimination challenges in clear areas

#### System sensitivity

System sensitivity reduction was participants’ major concern. The reduced system sensitivity is described as the lower accuracy of the health system and HSPs in case of identification of suspected malaria cases. This is due to very low malaria transmission in the elimination phase. In recent years, malaria cases were absent and MCM or readiness for ME was ignored, particularly, no local transmission has been reported in East Azerbaijan Province for 15 years. It seems that the health system and HSPs pay less attention to malaria recently.

#### Possibility of re-introduction

Interviewees’ other concerns and challenges pertained to the possibility of malaria re-introduction in one or two counties of the province (Kaleybar) as well as the neighbourhood countries with endemic areas such as Pakistan and Afghanistan. Insecticide resistance, swamp areas and underdeveloped agricultural land in some places were raised as other concerns.

#### Exiting febrile suspected cases from system surveillance chain

One of the participants emphasized that in the current health system condition, there existed many significant clinics in the private sector that could provide medical care for malaria cases without appropriate MCM or sending any report. Needless to say that MCM is acceptable only when it is done by the public sector and zero reporting does not imply there is no malaria case. In this regard, participants were in the opinion that effective coordination between private and public sectors were required up until the time that, no suspected cases exited from system surveillance.

## Discussion

The present study investigated 18 years trend of malaria cases and indigenous transmission in a clear area (East Azerbaijan Province), compared with whole country status. The findings revealed that malaria incidence was seriously reduced with no occurrence of indigenous transmission from 2005 to 2018 (14 years), in other words, province has been in the elimination phase for many years. In Iran, also, for the first time in 2018, indigenous transmission was not reported and it is considered a great success for the national ME goal.

In Iran, malaria pre-elimination programme started in 2009 with WHO’s technical support [8]. This programme was aimed to interrupt *Plasmodium falciparum* transmission by the end of 2015 and to achieve a malaria-free country in 2025. During the past decades, vector control and other interventions were devised and implemented with the political commitment for malaria control and pre-elimination. Surprisingly, in the last 2 years (2018 and 2019), the number of indigenous cases at the national level has reached to zero [7]. In Iran, passive and active case finding among suspected cases with relevant symptoms and epidemiological history such as fever and travel history applied in the current malaria surveillance system. The case finding in Iran focuses on passive case detection. By definition, if a positive case is found, active case detection of the patient’s surroundings is performed by the community health workers (*Behvarz* in Persian) or volunteers by taking blood smears (slides) and RDTs. Laboratory personnel continuously examined microscopic slides for malaria diagnosis. Standard operating procedures were performed for quality assurance of malaria diagnostic tests on all positive and 10% of negative slides [17, 18]. Initiated in 2009 in Iran, RDTs are ordinarily used when microscopic detection is not available, in outlying areas (> 50 km from malaria diagnostic centers), outbreaks and among migrants [18]. Using this process by trained volunteers in rural areas can play a critical role in early case finding. The polymerase chain reaction (PCR) is used when there is a discrepancy between microscopic slides and RDTs’ tests results or when both are negative; nonetheless, the clinical and epidemiological symptoms are in favour of malaria [7, 19].

In the last decades, many effective efforts performed to control malaria and to get closer to elimination criteria in Iran. To name a few as the most important strategies, expanded coverage of integrated vector management (IVM), urgent report system, appropriate case management, training of microscopists, developing a malaria early warning system, rural malaria mobile teams, and enhanced inter-sectoral collaborations are mentioned [7, 9, 20].

After the ME programme’s national launch in 2009, this programme was strongly reinforced. Consequently, effective vector control interventions were implemented such as distribution of free insecticide-treated nets (ITN), periodic indoor residual spraying (IRS), insecticide resistance monitoring, using various larvicides, sibling species, molecular study, use of plants for larval control, using bed nets and long-lasting impregnated nets, using RDTs, development of environmental and socio-economic status, including water pipe network, better housing, health and welfare amenities [9, 21–23].

Kyrgyzstan, Sri Lanka, and Paraguay are countries that have recently obtained the WHO certificate of malaria elimination while the UAE is the first country to obtain that in the Middle East, [2]. In India, by streamline programme planning, management, and improvement malaria surveillance system, approximately the same strategies as the Iranian health system were utilized to achieve the goal of malaria elimination by 2027, [24]. In Saudi Arabia, the malaria control programme initiated in 1948 and some major achievements were made in this respect. At the moment, this programme is focused on imported cases, *P. falciparum* resistance to chloroquine, border malaria, and vector resistance to insecticides [25].

In China, the malaria elimination programme was launched in 2010 to achieve zero indigenous cases in the country by 2015 and to eliminate by 2020. To achieve malaria elimination phase, specific requirements such as timely malaria case detection, timely surveillance, response to all malaria cases, treatment, anti-mosquito interventions, strengthening people’s awareness, and capacity building for malaria diagnosis were assigned [26].

Despite these remarkable accomplishments, there are challenges to achieve the goal of elimination. However, in clear areas, the main concern is pertinent to system sensitivity reduction and inappropriate MCM. While several research studies were conducted regarding malaria trends and situation in Iran and endemic areas [7, 14, 27] a few were carried out in clear areas and investigated their challenges and readiness for ME compared with the trend of malaria at the national level [28]. Moreover, in this study, PHC surveillance system in malaria identification and management were assessed between 2004 and 2018.

The findings indicated that the screening rate (by slides) from febrile suspected cases decreased in contrast to slide reading in less than 48 h period (from the time slide was taken until the microscopic examination was adjusted). It is noteworthy that most of the slides were prepared and tested in this interval time in the last years.

As stated before, East Azerbaijan is a clear area of malaria which is in the elimination phase. All 7 malaria cases in the last 10 years were imported cases. Effective efforts, political commitment, and improvement in identifying and treating suspected cases were emphasized and implemented by national and provincial malaria control programme.

It is very vital for policymakers and health systems to obtain up-to-date information at local level for better understanding of how to meet ME criteria and ascertain an appropriate strategy for adjusting barriers and challenges. In-depth interviews with heath managers, malaria control programme experts and researchers elicited many novel viewpoints that could be considered as key messages to fulfill ME criteria in clear areas and countries in elimination phase.

### HSPs readiness

The majority of participants were in the opinion that in clear areas appropriate MCM status was unknown and more attention was required. They added that the ideal way to demonstrate HSPs readiness and performance, is to get benefit from role playing technique through changing one’s behaviour to pretend to be a suspected malaria case. Hence, it can keep track of HSPs performance in a real way. HSPs ought to assess all febrile patients and outpatients systematically in terms of malaria morbidity risk. Similarly, this issue was raised in other studies from endemic countries and low transmission areas while few studies from clear areas are available [13, 28–30], although there exist globally serious defects in correct MCM [12, 31, 32]. To put it another way, it is urgently required that interventions for appropriate MCM, especially, in the private sector be applied.

As a matter of fact, in low transmission areas and elimination phases as well as the investigated province, KAP studies, particularly, to assess HSPs performance and practice in role-playing situation are very low and in this province, no study was conducted. Perhaps, this might be due to the absence of malaria cases. Health policymakers need to realize the effect of their efforts and many aspects of the national malaria elimination programme situation as well as ME criteria. Therefore, the current study could be considered as a key path for ME. However, in order to obtain accurate and realistic information on the status and readiness of areas in the elimination phase, community-based studies are required.

Appropriateness of the rate of identification of malaria from febrile or suspected cases was another point expressed by the interviewees; whereas, the quality of slides seems low in the first line of referral system. By definition, most of these slides were taken by the first line HSPs in PHC and mostly in rural areas. As malaria cases were rare in the elimination phase, thus, the quality of slides was lower. It is true that malaria RDTs are utilized, however, according to participants’ opinions, in places with higher febrile patient flow (e.g. Emergency wards) RTDs have to be emphasized and utilized more. In this regard, intensified monitoring and evaluation of all components of the health system, sentinel surveillance, financial support, evidence-based research, community engagement, and intersectional collaboration are essential elements to be assigned [33]. In the same way, a study from a low transmission area recommended that a positive feedback mechanism should be considered for HSPs based on their quality of performance to health services [3].

### Possible challenges of clear areas in elimination phase

Although a few participants declared that there was no concern and challenge in ME in the province, many of them emphasized the possible existing challenges on the way to achieve the elimination criterion required to be improved by the health system.

One noteworthy finding that emerged from the interviews was related to the notion of decreasing system sensitivity in East Azerbaijan Province and in other clear areas which was considered as the most significant challenge for ME. It might be because malaria cases were seriously reduced and HSPs and health facility attendance for malaria and suspected cases were diminished. Furthermore, many managers and policymakers focused on non-communicable diseases. However, very few studies were conducted and the relation between decreasing system sensitivity and malaria identification and case management were poorly understood. Some pieces of evidence support this notion [34, 35].

Another point raised by participants was regarding the possibility of malaria re-introduction in the province due to two reasons; first, in some counties of the province, population are prone to malaria morbidity where weather and geographical conditions facilitate transmission of this vector-borne disease. Second, the existence of malaria-endemic countries in the region and the possibility of entrance by cases affected by malaria from Pakistan and Afghanistan; however, fortunately, illegal travellers from endemic countries to East Azerbaijan Province are rare.

One of the participants believed that in very low transmission areas, it was probable that febrile cases were withdrawn from the malaria surveillance system. Despite HSPs readiness, it is acceptable for ME that suspected cases or febrile returning travellers directly refer to the pharmacy and clinicians in the private sector and receive anti-fever drugs or be treated for other common related diseases in the region. Globally, although this issue is a major concern in order to achieve elimination, it is underestimated in the elimination phase. Another striking issue is pertinent to establishing coherence between public and private sectors in managing the patients with suspected malaria. No patient is willing to subject herself/himself to the inconvenience of a crowded public sector with failures in service providing and low quality of services. Thus, some patients seek their medical needs in the private sector.

## Conclusion

The current study explored 18 years trend of malaria and its situation in East Azerbaijan Province which is in the elimination phase. In the last decade in this province, 7 malaria cases were recorded which all were imported cases, however, this province enjoys much better conditions compared with national status.

Based on the interviews conducted with health managers, malaria field experts and researchers, there exist many challenges and gaps in HSPs readiness and correct MCM in the elimination phase. The key message of this research study is that global health systems, especially, areas with an absence of malaria incidence or very low malaria transmission must be alert bearing in mind that zero reporting and no local transmission do not guarantee for ME.

It is worth noting that these findings are based on interviews and the opinions of individuals, so for better and real understanding and recognition from HSPs readiness and challenges in elimination phase, holistic and role-playing evidence-based studies are recommended to assess HSPs practice in MCM in clear areas and health system readiness as well as performance for ME.

## Data Availability

Part of the data in this manuscript were qualitative and derived from in-depth interviews. Another part was obtained from aggregated and prepared data but it is available from the corresponding author on a reasonable request.

## Abbreviations

MCM: Malaria case management
ME: Malaria elimination
HSPs: Health service providers
PHC: Primary health care
WHO: World Health Organization
